# The Polyphenol-Rich Extract from *Psiloxylon mauritianum,* an Endemic Medicinal Plant from Reunion Island, Inhibits the Early Stages of Dengue and Zika Virus Infection

**DOI:** 10.3390/ijms20081860

**Published:** 2019-04-15

**Authors:** Elodie Clain, Juliano G. Haddad, Andrea C. Koishi, Laura Sinigaglia, Walid Rachidi, Philippe Desprès, Claudia N. Duarte dos Santos, Pascale Guiraud, Nolwenn Jouvenet, Chaker El Kalamouni

**Affiliations:** 1 Université de la Réunion, INSERM U1187, CNRS UMR 9192, IRD UMR 249, Unité Mixte Processus Infectieux en Milieu Insulaire Tropical, Plateforme Technologique CYROI, Sainte Clotilde, 97490 La Réunion, France; marie-elodie.clain@univ-reunion.fr (E.C.); juliano.haddad@univ-reunion.fr (J.G.H.); philippe.despres@univ-reunion.fr (P.D.); pascale.guiraud@univ-reunion.fr (P.G.); 2 Laboratorio de Virologia Molecular, Instituto Carlos Chagas, ICC/FIOCRUZ/PR, Curitiba, Parana 81350-010, Brazil; ackoishi@gmail.co (A.C.K.); clsantos@fiocruz.br (C.N.D.d.S.); 3 Viral Genomics and Vaccination Unit, UMR CNRS 3569, Institut Pasteur, 75724 Paris, France; laura.sinigagli@gmail.com (L.S.); nolwenn.jouvenet@pasteur.fr (N.J.); 4 Université Paris Diderot, Sorbonne Paris Cité, 75013 Paris, France; 5 Université Grenoble Alpes, SYMMES/CIBEST UMR 5819 UGA-CNRS-CEA, INAC/CEA, 38000 Grenoble, France; Walid.rachidi@univ-grenoble-alpes.fr

**Keywords:** Zika virus, Dengue virus, antiviral activity, natural compounds, nutraceuticals, *Psiloxylon mauritianum*

## Abstract

The recent emergence and re-emergence of viral infections transmitted by vectors, such as the Zika virus (ZIKV) and Dengue virus (DENV), is a cause for international concern. These highly pathogenic arboviruses represent a serious health burden in tropical and subtropical areas of the world. Despite the high morbidity and mortality associated with these viral infections, antiviral therapies are missing. Medicinal plants have been widely used to treat various infectious diseases since millenaries. Several compounds extracted from plants exhibit potent effects against viruses in vitro, calling for further investigations regarding their efficacy as antiviral drugs. Here, we demonstrate that an extract from *Psiloxylon mauritianum*, an endemic medicinal plant from Reunion Island, inhibits the infection of ZIKV in vitro without exhibiting cytotoxic effects. The extract was active against different ZIKV African and Asian strains, including an epidemic one. Time-of-drug-addition assays revealed that the *P. mauritianum* extract interfered with the attachment of the viral particles to the host cells. Importantly, the *P. mauritianum* extract was also able to prevent the infection of human cells by four dengue virus serotypes. Due to its potency and ability to target ZIKV and DENV particles, *P. mauritianum* may be of value for identifying and characterizing antiviral compounds to fight medically-important flaviviruses.

## 1. Introduction

The Zika virus (ZIKV) and Dengue virus (DENV) are mosquito-borne enveloped viruses belonging to the *Flavivirus* genus and constitute a worrisome threat to global human health [1,2]. The genome of flaviviruses consists of a positive-sense single stranded RNA of approximately 11 kb [3]. After the viral processing of the polyprotein, ten viral proteins are produced: three structural proteins (C, M and E) and seven nonstructural proteins (NS1, NS2A, NS2B, NS3, NS4A, NS4B and NS5) [4]. The E protein mediates the attachment of the viral particles to the host cell membrane [5]. This step is necessary for the establishment of the infection [6,7].

Dengue fever, caused by 4 viral serotypes (DENV 1–4), is the most prevalent mosquito-borne viral infection in tropical and subtropical countries, including the southwest of the Indian Ocean region [2]. An epidemic of DENV-2 is currently taking place in the French overseas region Reunion Island. ZIKV causes important outbreaks in the South Pacific, Americas and South-East Asia. The recent ZIKV epidemics in French Polynesia and South America were associated with severe fetal brain injury and neurological defects in adults such as the Guillain-Barré syndrome [8,9,10,11]. ZIKV infection is now identified as a sexually transmitted illness as well [12,13]. Phylogenetic studies allowed the division of ZIKV into African and Asian lineages [14]. Since 2007, the leading cause of major epidemics is the ZIKV Asian genotype [15].

Medicinal plants, which have been used as a treatment or prevention against several human infectious diseases for millenaries, remain a remarkable potential source of antiviral compounds against flaviviruses [16,17]. The Reunion Island, which belongs to the Mascarene Archipelago, is classified as a biodiversity hotspot due to its exceptional flora and endemic plants. These could provide active phytochemicals against viral infections [18,19,20]. Phytochemicals, including alkaloids, coumarins, flavonoids, terpenoids, polyphenolics and saponins, have already been described for their antiviral activity against numerous enveloped RNA viruses including flaviviruses [17,21,22,23]. Indeed, the polyphenols epigallocatechin gallate (EGCG) from green tea, delphinidin, and curcumin, have been found to inhibit ZIKV and DENV infection [24,25,26,27]. We recently reported that a polyphenol-rich extract from *Aphloia theiformis*, an indigenous medicinal plant from Reunion Island, prevents ZIKV and DENV infection through an inhibition of the virus entry steps [28]. *Psiloxylon mauritianum*, an endemic medicinal plant recently listed in the French pharmacopoeia, has been extensively used in folk medicine among the local people of the Mascarene Islands in the Indian Ocean [29,30]. Given the high content of polyphenols in a *P. mauritianum* extract, we decided to evaluate its ability to inhibit ZIKV and DENV infection. In the present study, we showed that *P. mauritianum* severely restricts the binding of virus particles to the host-cells.

## 2. Results

### 2.1. Psiloxylon Mauritianum Extract Did Not Induce Genotoxicity in Human Cells

**Figure 1:**
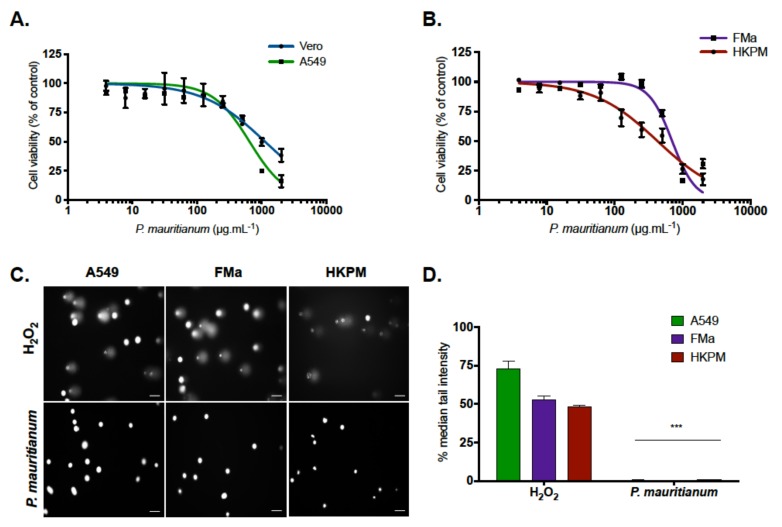
The cytotoxicity and genotoxicity of the *P. mauritianum* extract on mammalian cells. (**A**) Vero and A549 cells were incubated with different concentrations of the *P. mauritianum* extract for 24 h. The cell viability was determined using the metabolic activity by an MTT assay. (**B**) Human primary keratinocytes (HKPM) and fibroblasts (FMa) were treated with an increased concentration of the *P. mauritianum* extract for 24 h, and their viability was evaluated by an MTT assay. (**A**,**B**) The results are the means ± SD of three independent experiments and are expressed as the relative value compared to untreated cells. (**C**) Human cells (A549, HKPM and FMa) were treated 24 h with 100 µg mL^−1^ of the *P. mauritianum* extract. The cells were treated 10 min with H_2_O_2_ (50 µM) as a positive control. The genotoxicity was determined by observation of DNA degradation using a COMET assay visualized by Gel Red. The images are representative of three independent experiments. Scale bars are 50 µm. (**D**) The quantification of the COMET signal from the experiments represented in the images by using a COMET assay IV software. The results are means ± SD of three independent experiments and are expressed as the percentage of the tail intensity. A one-way ANOVA and Dunnett’s test for multiple comparisons were used for the statistical analysis (*** *p* < 0.001).

Prior to evaluating the potential antiviral activity of the *Psiloxylon mauritianum* extract, we determined whether it exhibited cytotoxic and genotoxic effects on different mammalian cell lines, using MTT and COMET assays, respectively. The cells were treated with different concentrations of the *P. mauritianum* extract ranging from 4 to 2000 µg mL^−1^. The results showed a concentration-dependent toxicity with a cell viability beginning to decrease starting at concentrations higher than 250 µg mL^−1^. The cytotoxic concentration that inhibited 50% of the cell viability (CC_50_) was 1044 ± 106.2 µg mL^−1^ and 657 ± 15.7 µg mL^−1^, for Vero and A549 cells, respectively (Figure 1A). We also determined the CC_50_ on human primary keratinocytes (HKPM) and fibroblast (FMa) cells. Figure 1B shows dose-response curves, with a CC_50_ for HKPM and FMa of 353 ± 84.4 µg mL^−1^ and 820 ± 26.5 µg mL^−1^, respectively (Figure 1B). We next determined the maximal noncytotoxic concentration (MNTC) on different mammalian cell lines, using a nonlinear regression curve. The concentration of 100 µg mL^−1^ of the *P. mauritianum* extract, that maintained 90% of Vero and A549 cell viability (Figure 1A), was chosen to test the potential antiviral activity of the *P. mauritianum* extract on Vero cells.

To further characterize the toxicity of the *P. mauritianum* extract, we analyzed human cell DNA damage by a COMET assay. A549 cells were treated with a maximal noncytotoxic concentration (MNTC) of *P. mauritianum* (100 µg mL^−1^) for 24 h, and their DNA degradation was then analyzed with a COMET assay. H_2_O_2_, which is known to induce DNA degradation [31], was used as a positive control. No comet formation and no DNA degradation were observed in the cells treated with the *P. mauritianum* extract (Figure 1C,D). However, the COMETs were clearly visible when the cells were treated with the positive control H_2_O_2_ (Figure 1C,D). We obtained the same results using a cytotoxic concentration (500 µg mL^−1^) of the *P. mauritianum* extract on A549 cells (Figure S1).

These results suggest that the *P. mauritianum* extract did not induce human cell DNA degradation, even at a cytotoxic concentration.

### 2.2. P. mauritianum Extract Inhibits ZIKV Attachment to Mammalian Cell Surface

**Figure 2:**
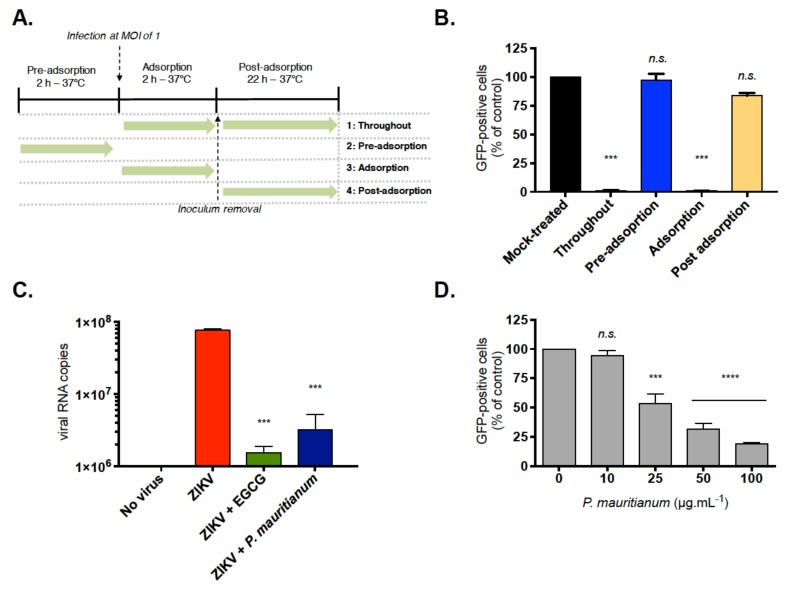
*P. mauritianum* extract inhibits ZIKV attachment to the cell surface. (**A**) Schematic representation of time-of-drug-addition assays used to characterize the antiviral activity of the *P. mauritianum* extract (100 µg mL^−1^). Green arrow indicates the presence of the plant extract. (**B**) Flow cytometric analysis of GFP expression in Vero cells infected with ZIKV^GFP^ during 24 h at MOI of 1 under the different experimental conditions shown in (**A**). The results are means ± SD of three independent experiments and are expressed as the relative value compared to untreated infected cells. (**C**) Virus binding assays: Vero cells were infected with ZIKV-MR766 at MOI of 1 for 1 h at 4 °C with or without 100 µg mL^−1^ of the *P. mauritianum* extract. EGCG (100 µM) were used as a positive control. The number of virus particles bound to the cell surface was measured by RT-qPCR. The values represent the mean ± SD of three independent experiments. (**D**) Viral inactivation assays: Vero cells were infected with ZIKV^GFP^ pre-incubated during 1 h at 37 °C with four different concentration of *P. mauritianum* extract. A flow cytometric analysis of GFP fluorescence was performed 24 hp.i. The results shown are means ± SD of three independent experiments and are expressed as the relative value compared to untreated infected cells. Statistical analyses were performed using a one-way ANOVA and Dunnett’s test for multiple comparisons (*** *p* < 0.001; **** *p* < 0.0001; *n.s* = not significant).

To assess the antiviral activity and determine which steps of viral replication were affected by the *P. mauritianum* extract, time-of-drug-addition assays were performed in Vero cells (Figure 2A), as previously described [28], using a chimeric molecular clone of the ZIKV strain MR766 expressing a GFP reporter gene (ZIKV^GFP^) [32]. This virus allows the monitoring of viral replication by assessing GFP fluorescence by flow cytometry. The ‘pre-adsorption’ experimental condition provides an insight into the ability of the plant extract to prevent a ZIKV infection by potentially interacting with ZIKV receptors or inducing an immuno-stimulating effect. The ‘adsorption’ experimental condition evaluates the capacity of the plant extract to interfere with the internalization process of the viral particles into the host-cell. The ‘post-adsorption’ experimental condition determines whether the extract is efficient at inhibiting the intracellular replication and polyprotein processing/synthesis of ZIKV [4]. When 100 µg mL^−1^ of *P. mauritianum* extract was added throughout the experiment and concomitantly to the virus for 2 h (adsorption), the fluorescence intensity representing the viral replication was detected only in 2% of cells (Figure 2B). By contrast, no effect was observed when the *P. mauritianum* extract was added at pre-adsorption or 2 h post-adsorption (Figure 2B). These results suggest that the antiviral activity of the *P. mauritianum* extract was not mediated by the inhibition of the viral protein production but rather by the inhibition of early steps of the viral replication, possibly viral entry mechanisms.

The early steps of the viral infection include binding to the cell surface and internalization via endocytosis. To determine which of these steps are targeted by the *P. mauritianum* extract, viruses generated with the ZIKV-MR766 strain molecular clone were incubated on Vero cells for 1 h at 4 °C, to allow the attachment to the cell surface and prevent viral internalization, in the presence or absence of the *P. mauritianum* extract (100 µg mL^−1^). The amount of attached ZIKV particles was determined by RT-qPCR. EGCG, which is known to inhibit the ZIKV attachment [27,28,33], was used as a positive control. Our data show that 10 times less viral particles were attached to the surface of the cell membrane in the presence of the *P. mauritianum* extract, as compared to the untreated control cells (Figure 2C). Thus, the treatment with the *P. mauritianum* extract significantly affects ZIKV binding to the cell surface (Figure 2C).

We also performed a viral inactivation assay to determine if the *P. mauritianum* extract neutralizes ZIKV infectivity. Briefly, ZIKV^GFP^ particles were incubated for 1 h at 37 °C with different concentrations of plant extract, prior to infection [28]. At 24 h post-infection, a significant decrease of GFP-positive cells (up to 80%) was observed in cells treated with 100 µg mL^−1^ of the plant extract, as compared to untreated ones (Figure 2D). Using a nonlinear regression curve, the concentration of *P. mauritianum* that inhibits 50% of ZIKV infection (IC_50_) was estimated at 19.5 ± 4.8 µg mL^−1^. Based on the cytotoxicity and the antiviral activity, the selectivity index (SI = CC_50_/IC_50_) was then calculated. The SI of *P. mauritianum* was 53.5. Together, these results suggest that the antiviral activity of the *P. mauritianum* extract is mediated by an interaction between the extract and ZIKV particles.

### 2.3. P. mauritianum Extract Inhibits Infection of African and Asian Lineages of ZIKV

To further validate the potential anti-ZIKV activities of the *P. mauritianum* extract, the African lineage strain ZIKV-MR766 was incubated with the *P. mauritianum* extract for 1 h at 37 °C prior to the Vero cells infection through the viral inactivation assay. Several methods were used to assess the viral infection: the viral replication was evaluated by the presence of single-stranded viral RNA (ssRNA) using fluorescence in situ hybridisation (FISH)-based assays, the viral protein production was quantified by immunofluorescence assays and the viral production was measured by plaque forming assays.

**Figure 3:**
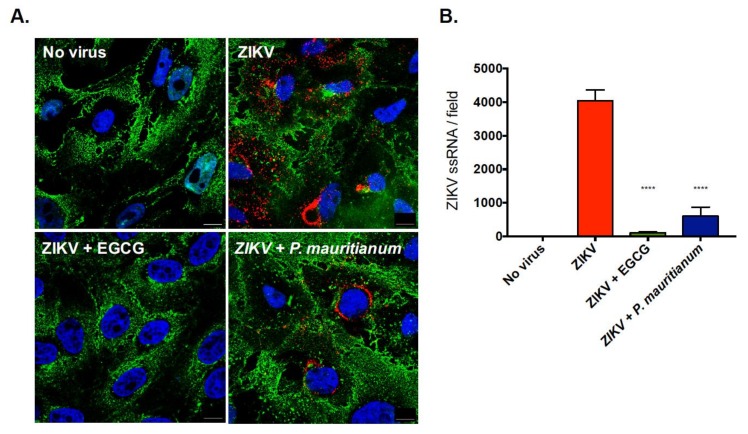
*P. mauritianum* extract inhibits the production of ZIKV ssRNA. ZIKV-MR766 particles were incubated with the *P. mauritianum* extract for 1 h at 37 °C. The EGCG treatment (100 µM) was used as positive control. Vero cells were left uninfected or were infected at an MOI of 1 for 24 h. (**A**) Cells were processed for FISH using a probe specific for viral RNA (red) and then stained with NucBlue to visualize the nuclei (blue). Cell membranes were stained with AF488-conjugated wheat germ agglutinin. Images are representative of three independent experiments. Scale bars are 20 μm. (**B**) Quantification of ZIKV ssRNA spots counted per field (fields contained on average 300 cells) from the experiments represented in the images. Data are means ± SD of three independent experiments. Statistical analyses were performed using a one-way ANOVA and Dunnett’s test for multiple comparisons (**** *p* < 0.0001).

The production of ssRNA was first measured by FISH coupled to confocal microscopy 24 h post-infection (hp.i). The cells treated with EGCG, which is known to inhibit ZIKV entry [26,28,33], were used as positive controls. The FISH probe produced no signal in the non-infected control cells and a bright signal in the cytoplasm of the infected cells (Figure 3A), validating its specificity. To better visualize the edge of the cells and the localisation of ssRNA, a fluorescence dye binding to cellular membranes was included in the analysis. The incubation of ZIKV-MR766 with the *P. mauritianum* extract resulted in a significant reduction of the number of ssRNA molecules per field, as compared to mock-treated infected cells (Figure 3B).

**Figure 4:**
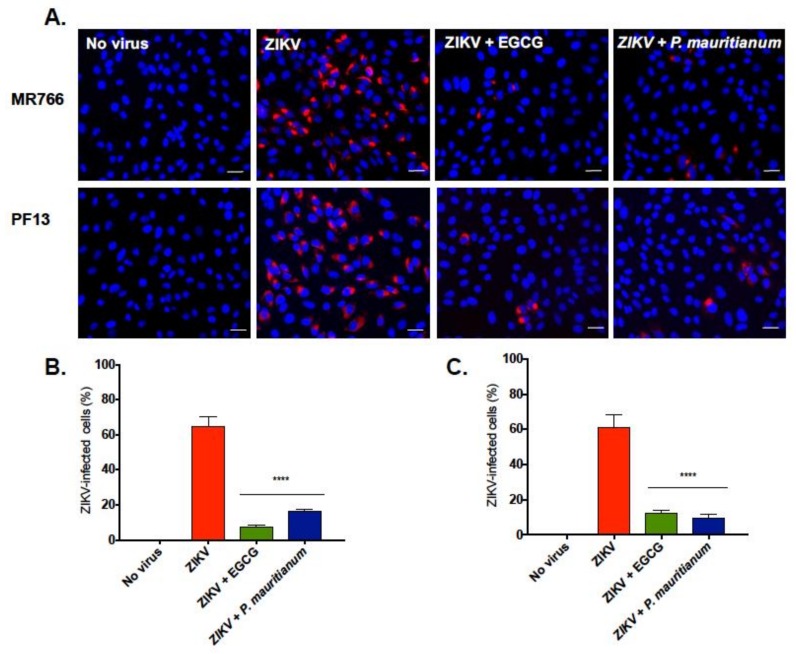
Antiviral activity of the *P. mauritianum* extract against the African and Asian ZIKV lineages. ZIKV-MR766 or ZIKV-PF13 were incubated with the *P. mauritianum* extract for 1 h at 37 °C. EGCG (100 µM) was used as a positive control. The Vero cells were infected at a MOI of 1 for 24 h. (**A**) The cells were processed for an immunofluorescence assay. The ZIKV E protein (red) and nuclei (blue) were visualized by fluorescence microscopy. The images are representatives of three independent experiments. Scale bars are 50 µm. The quantification of the number of (**B**) ZIKV-MR766 and (**C**) ZIKV-PF13 infected Vero cells from the experiments are represented in the images. The results shown are the means ± SD of three independent experiments. Statistical analyses were performed using a one-way ANOVA and Dunnett’s test for multiple comparisons (**** *p* < 0.0001).

In parallel, the viral protein production was evaluated 24 hp.i by immunofluorescence assays, using an antibody that recognizes the viral protein E. The EGCG treatment was used as a positive control. To determine whether the *P. mauritianum* extract exhibited an antiviral activity against another viral strain, the contemporary Asian lineage ZIKV-PF13, which was responsible for the epidemic in 2013 in French Polynesia, was included in the analysis [34]. The production of the viral protein E of both strains was affected by 100 µg mL^−1^ of the *P. mauritianum* extract (Figure 4A). The percentage of ZIKV-infected cells was reduced to 16% and 9% in the presence of *P. mauritianum*, upon infection with ZIKV-MR766 and ZIKV-PF13, respectively (Figure 4B,C). By contrast, up to 60% of the non-treated cells were infected (Figure 4B,C). The extract was as potent as EGCG in inhibiting the ZIKV infection (Figure 4B,C). In this assay, ZIKV-PF13 was as sensitive as the African lineage to the *P. mauritianum* treatment.

**Figure 5:**
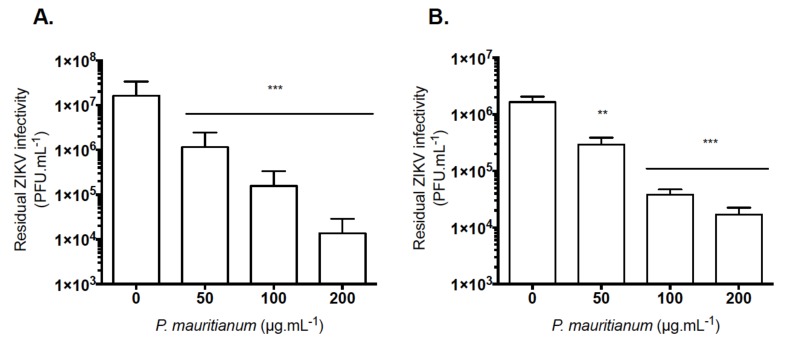
The *P. mauritianum* extract reduces the viral progeny production of the African and Asian ZIKV lineages. (**A**) ZIKV-MR766 or (**B**) ZIKV-PF13 were incubated with different concentrations of the *P. mauritianum* extract for 1 h at 37 °C. The Vero cells were infected at a MOI of 1 for 24 h. The release of infectious viral particles was measured by plaque forming assays. The results shown are the means ± SD of three independent experiments. Statistical analyses were performed using a one-way ANOVA and Dunnett’s test for multiple comparisons (** *p* < 0.01; *** *p* < 0.001).

Finally, plaque forming assays confirmed that both strains were sensitive to the *P. mauritianum* extract (Figure 5A,B). At the maximal noncytotoxic concentration of 100 µg mL^−1^, *P. mauritianum* decreased up to 2-log the production of infectious viral particles (Figure 5A,B). ZIKV-MR766 appeared to be slightly more sensitive than ZIKV-PF13 to the *P. mauritianum* extract.

### 2.4. P. mauritianum Extract Exerts Antiviral Activity against Four DENV Serotypes

We next wondered if the *P. mauritianum* extract exerts an antiviral activity against DENV, another medically-relevant flavivirus. The anti-DENV activity of the *P. mauritianum* extract was evaluated using four different serotypes (clinical isolates for DENV1-3 and a DENV-4 lab-adapted strain). The treatment with IFN-α 2A, which is known to block DENV replication [35], was used as a positive control. The *P. mauritianum* extract (100 µg mL^−1^) was pre-incubated with the four different serotypes of DENV for 1 h at 37 °C prior to infection. The human hepatoma cell lines Huh7.5, which are extensively used in Flaviviridae research and relevant for DENV infection [36,37], were used to perform these experiments. The cytotoxicity effect of the *P. mauritianum* extract on Huh7.5 cells was first evaluated using MTT assays. The concentration of 100 µg mL^−1^ of *P. mauritianum* did not affect the cell viability after 48 h of treatment (Figure S2).

**Figure 6:**
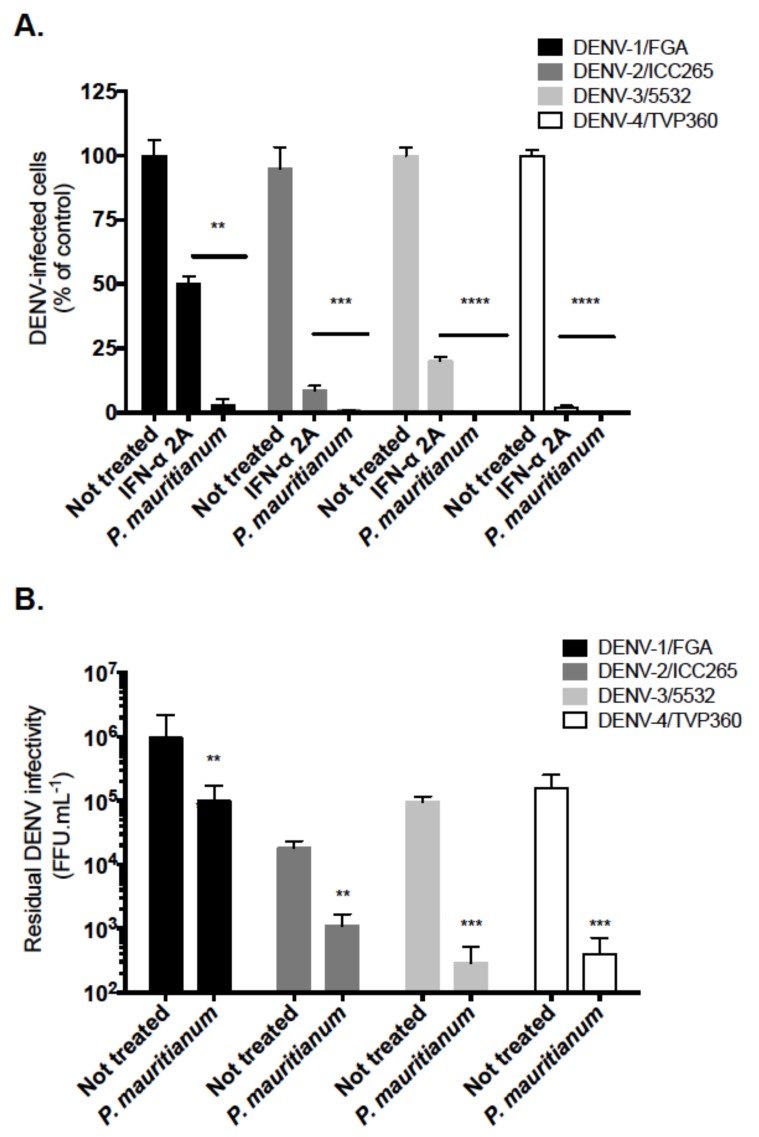
The *P. mauritianum* extract inhibits the infection of four DENV clinical isolates representative of the four serotypes. Four DENV serotypes (DENV-1, 2, 3 and 4) were pre-incubated with the *P. mauritianum* extract for 1 h at 37 °C. (**A**) Huh7.5 cells were infected for 48 h with DENV at the different MOI 0.2, 2, 0.5 and 2 for DENV 1–4, respectively. Recombinant IFN-α 2A (200 IU mL^−1^) was added 2 h post-infection and used as a positive control. The percentage of E positive cells was evaluated using the Operetta High-Content Imaging System (PerkinElmer). The results are the means ± SD of three independent experiments. (**B**) The residual infectious particles were titrated in C6/36 cells using a foci-forming immunodetection assay. The data represent the means ± SD from three independent experiments. A one-way ANOVA and Dunnett’s test for multiple comparisons were used for the statistical analysis (** *p* < 0.01; *** *p* < 0.001; **** *p* < 0.0001).

The number of E-positive cells were quantified at 48 hp.i using antibodies against the E protein and the Operetta High-Content Imaging System (PerkinElmer). The *P. mauritianum* extract significantly decreased the number of E-positive cells (Figure 6A). DENV-3 and -4 appeared more sensitive to the *P. mauritianum* action than DENV-1 and -2 (Figure 6A). The production of infectious viral particles was then quantified using foci forming immunodetection assays to relate the reduction of the number of E-positive cells to the DENV infectivity. A decrease of up to 2 log of the DENV infectivity was observed upon DENV-3 and -4 infections when the virions were treated with 100 µg mL^−1^ of the *P. mauritianum* extract (Figure 6B). These results show that the *P. mauritianum* extract exhibits potent antiviral activity against the four DENV serotypes.

## 3. Discussion

Despite the recent ZIKV emergence worldwide and its subsequent classification as a major public health problem by the World Health Organization in 2016 [1], there are still no vaccine or licensed antivirals. Thus, the use of medicinal plants as a simple and economical means of treating the viral infection merits investigations. In this study, we evaluated the antiviral activity of *Psiloxylon mauritianum* extract against ZIKV and DENV. Our data demonstrate that non-toxic concentrations of *P. mauritianum* (100 µg mL^−1^) significantly reduced the ZIKV infection in Vero cells, regardless of the ZIKV strains tested (historic and epidemic strains). We also demonstrated that *P. mauritianum* extract prevents the infection of human cells by four DENV serotypes. We showed that *P. mauritianum* extract acts on early steps of the viral replication cycle, in a similar way than EGCG, a green tea polyphenol, which is known to inhibit viral entry into the host cells [27,28,33]. Our attachment and viral inactivation assays show that *P. mauritianum* extract inhibits the viral attachment to the cell surface, probably via a direct interaction with the outer membrane of ZIKV particles. This type of interaction was previously described between EGCG and ZIKV or DENV particles [26,27,28]. We propose that the active compounds of *P. mauritianum* extract act through a similar mechanism, probably via binding to the surface of the virions.

*P. mauritianum* is an endemic medicinal plant of the Mascarene Islands, more specifically of the Reunion and Mauritius Islands. The folk use of this plant has been described for the treatment and management of amenorrhea, dysentery and common infectious diseases [29,38,39]. The high amount of polyphenols of this endemic medicinal plant provides a significant antioxidant activity [39]. Our phytochemical analysis of *P. mauritianum* extract, performed with an ultra-high-performance liquid chromatography-diode array detector coupled to mass spectrometry (UHPLC-DAD-MS), confirmed the phenolic-richness composition of this plant, mainly a glycosylated derivate of gallic acid, quercetin and kaempferol (Figure S3 and Table S1). Indeed, numerous polyphenol compounds have been described for their anti-flavivirus activities, such as EGCG, which is also efficient against a broad spectrum of enveloped RNA and DNA viruses [27,33,40,41,42,43,44], delphinidin [27], naringenin [35], isoquercitrin [23] and curcumin [25]. Likewise, we have recently shown that another polyphenol-rich extract from Reunion Island medicinal plant *Aphloia theiformis* inhibits ZIKV and DENV infection [28]. Thus, it would be of great interest to identify the phytochemicals responsible for *P. mauritianum* and *A. theiformis* antiviral activities or whether there is a synergetic effect of several compounds leading to antiviral activity.

Importantly, in addition to this antiviral activity, we demonstrated that *P. mauritianum* extract did not exert a genotoxic effect on several mammalian cells, including human primary cells relevant for arboviral infection. Our COMET assay showed that the plant extract did not cause any deleterious effect on the cell DNA. Previous studies assessing the genotoxicity of medicinal plants showed great variability in their genotoxic potential, depending on the plants and types of extracts tested [45,46,47,48]. We demonstrated that *P. mauritianum* did not exert a high cytotoxicity regardless of the cell lines tested. These non-deleterious effects further indicate that *P. mauritianum* extract is a potential candidate for the development of new antiviral drugs to fight ZIKV and DENV infections.

In conclusion, our data demonstrated that the extract from *Psiloxylon mauritianum*, an endemic medicinal plant from the Reunion and Mauritius Islands, exhibits an antiviral activity against historical and contemporary strains of ZIKV, as well as against four clinical isolates of DENV. Moreover, this plant extract does not exert a deleterious effect on mammalian cells, revealing that it could be an important source for antiviral drugs to treat Zika and Dengue fever in patients.

## 4. Materials and Methods

### 4.1. Cells, Viruses and Reagents

Vero cells (ATCC, CCL-81) and human-derived Huh7.5 hepatoma cells (ATCC, PTA-8561TM; Manassas, VA, USA) were cultured in an Eagle minimum essential medium (MEM) supplemented with a 5% heat-inactivated Fetal Bovine Serum (FBS), 2 mmol L^−1^ L-Glutamine, 1 mmol L^−1^ sodium pyruvate, 100 U mL^−1^ of penicillin, 0.1 mg mL^−1^ of streptomycin and 0.5 µg mL^−1^ of fungizone (Amphotericin B) (PAN Biotech, Aidenbach, Germany) under a 5% CO_2_ atmosphere at 37 °C. A549 cells (ATCC, CCL-185) were cultured in DMEM glutamax supplemented with 10% of FBS, 100 U mL^−1^ of penicillin, 0.1 mg mL^−1^ of streptomycin and 1% of non-essential amino acid (NEAA). Primary human keratinocytes plasty mammary (HKPM) and fibroblast (FMa) cultures were established by outgrowth from biopsies obtained immediately after breast plastic surgery from healthy donors, with their informed consent (Centre Hospitalier de Grenoble, Grenoble, France). Keratinocytes were cultured in Keratinocyte Serum-Free medium (KSFM) supplemented with 25 µg mL^−1^ Bovine Pituitary Extract (BPE), 1.5 ng mL^−1^ Epidermal Growth Factors (EGF) and 75 µg mL^−1^ primocin. C6/36 Aedes albopictus cells (ATCC CRL-1660, Manassas, VA, USA) were maintained at 27 °C in Leibovitz’s L-15 medium supplemented with 10% heat-inactivated FBS, 1% P/S, 1% MEM Non-Essential Amino Acids solution (Gibco/Invitrogen, Carlsbad, CA, USA) and 2% Tryptose Phosphate Browth (Gibco). The clinical isolate PF-25013-18 of ZIKV has been previously described [34,49]. ZIKV^MC-MR766NIID^ and the GFP-expressing strain of ZIKV^MC-MR766NIID^ (ZIKV^GFP^) are molecular clones of the ancestral strain MR766 of ZIKV [32]. DENV-1/FGA/89 was isolated in 1989 from a South American patient suffering from DF (GenBank: AF226687). DENV-2/ICC-265 was isolated from a DF patient in Brazil in 2009. DENV3/5532 was isolated in 2007 from a fatal case of dengue with visceral manifestations in a patient in Paraguay (GenBank: HG235027). DENV-4/TVP360 is a laboratory strain that was kindly provided by Dr. Ricardo Galler (Fundação Oswaldo Cruz, Rio de Janeiro, Brazil; GenBank: KU513442). DENV stocks were grown in C6/36 cells and titrated by a foci-forming immunodetection assay. Vero cells were infected with a MOI of 1. ZIKV strains were amplified on Vero cells. The anti-pan flavivirus E monoclonal antibody 4G2-Alexa Fluor 594 was purchased from RD Biotech.

### 4.2. Extraction of Plant Material

Fresh aerial parts of *Psiloxylon mauritianum* were collected in Reunion Island in 2016–2017. The voucher specimens (JF 1004) were deposited in the herbarium of the University of Reunion Island. A solvent-free microwave extraction was applied on *P. mauritianum* fresh aerial parts. After heating 500 g of plant material for 20 min at 800 W using an IDCO E200 extractor (IDCO SAS, Marseille, France), the extract was recovered by gravity. The aqueous extract was lyophilized using cryotec 20K (Cryotec, Saint-Gély-du-Fesc, France). The powder was then solubilized on sterile water and stored at −80 °C until used.

### 4.3. Cytotoxicity Assays

Two-fold dilutions of plant extract ranging from 4 to 2000 µg mL^−1^ were used to treat Vero, A549, HKPM and FMa cells at a density of 1×10^4^ cells per cultured well, in 96-well culture plates. The cells were rinsed with PBS 1×, and 120 µL of the culture medium mixed with 5 mg mL^−1^ MTT (3-[4-dimethylthiazol-2-yl]-2,5-diphenyltetrahzolium bromide) solution was added, after an incubation period of 24 h at 37 °C. Following an incubation of 2 h, the MTT medium was removed and the formazan crystals was solubilized with 100 µL of Dimethyl sulfoxide (DMSO). The absorbance was measured at 570 nm with a background subtraction at 690 nm. The CC_50_ was determined using a nonlinear regression on the Graphpad prism software (version 7.0; La Jola, CA, USA).

### 4.4. COMET Assays

A549, HKPM and FMa cells were seeded in 12-well plates one day before treatment. The cells were treated with different concentrations of plant extract during 24 h, then trypsinized, and kept in a freezing buffer until storage at −80 °C. Cell suspensions (100 µL) were mixed with 900 µL of 0.6% low melt agarose to obtained a density of 2×10^6^ cell mL^−1^. On agarose pre-coated slides, 100 µL of this suspension was put down and kept on ice for 10 min. A H_2_O_2_ (50 µM) treatment during 10 min was used as positive control. The slides were then brought in a lysis buffer for 1 h in the dark (pH 10, 2.5 M NaCl; 10 mM TRIS; 0.1 M EDTA; 1% Triton X-100; 10% DMSO). Following this, the slides were rinsed three times using a diluted Tris-HCl buffer (1.2 mM TRIS pH adjusted to pH 7.4 with concentrated HCl). Electrophoresis were performed on the slides for 30 min at 21 V in a cold denaturation buffer (0.3 M NaOH; 1 mM EDTA). After washing, the slides were stained with 50 µL of Gel Red (1:10000). The slides were observed using Zeiss Axioskop 2.0 with an Alled vision technologies camera (Stadtroda, Germany). The pictures were acquired and analyzed using the COMET assay IV software (Instem, Stone, UK).

### 4.5. Immunofluorescence and Flow Cytometry Assays

For the immunofluorescence assay, the cells were fixed (PFA, 3.7%) and permeabilized for 5 min (PBS 1× 0.15% Triton X-100) 24 hp.i. The cells were stained 1 h at room temperature in the dark for ZIKV using 4G2-Alexa Fluor 594 (1:1000 in PBS-BSA 2%). The DAPI staining was used to delineate the cells’ nuclei. Coverslips were mounted in Vectashield and the fluorescence was observed using a Nikon Eclipse E2000-U microscope. The Hamamatsu ORCA-ER camera and NIS-Element AR (Nikon) imaging software were used to capture images. For the flow cytometry assay, cells were harvested, followed by a fixation with paraformaldehyde 3.7% for 10 min. Then they were submitted to a flow cytometry analysis using CytoFLEX (Beckman Coulter, Brea, CA, USA). The results were analyzed using the Cytexpert software (Brea, CA, USA).

### 4.6. Virus Binding Assays

Virus binding assays were performed as previously described [23,28]. Briefly, Vero cells were seeded in 6-well plates at a density of 2 × 10^5^ cell per well. Pre-chilled cells were incubated with ZIKV-MR766 at MOI 1 with or without *P. mauritianum* extract for 1 h at 4 °C. Then, the virus input was removed and the cells were washed with cold MEM supplemented with 2% FBS. The samples were then submitted to RT-qPCR.

### 4.7. Virus Inactivation Assays

Virus inactivation assays were performed as previously described [23,28]. Briefly, ZIKV-MR766 or ZIKV-PF13 (2 × 10^5^ PFU) were mixed with *P. mauritianum* extract (100 µg mL^−1^) and then incubated for 1 h at 37 °C. Simultaneously, the same amount of virus was incubated with the medium without the plant extract as a control. The mixture (plant extract-virus) was then diluted 50-fold (final virus concentration, 1 PFU/cell) with MEM containing 2% FCS to yield a subtherapeutic concentration of plant extract, and the mixture was subsequently added to a Vero cell monolayer seeded in 6-well plates. As a comparison, ZIKV was mixed with *P. mauritanum*, diluted immediately to 50-fold without an incubation period and added to the Vero cells for infection. The 50-fold dilution served to titrate the plant extract below its effective dose and prevent meaningful interactions with the host cell surface. The inocula were discarded after 2 h of adsorption at 37 °C, the cells were washed with PBS twice and the incubation was maintained for 24 h at 37 °C before being subjected to cytometry or immunofluorescence assays as described above.

### 4.8. RT-qPCR

The total RNA, including genomic viral RNA, was extracted from cells with an RNeasy kit (Qiagen, Hilden, Germany) and reverse transcribed using an E reverse primer (5′-TTCACCTTGTGTTGGGC-3′) and M-MLV reverse transcriptase (Life Technologies, Villebon-sur-Yvette, France) at 42 °C for 50 min. A quantitative PCR was performed on a CFX96 Real-Time PCR Detection System (Applied Biosystems, Life Technologies, Villebon-sur-Yvette, France). Briefly, cDNA was amplified using 0.2 μM of each primer and GoTaq Master Mix (Promega, Charbonnières-les-bains, France). For each single-well amplification reaction, a threshold cycle (Ct) was calculated using the CFX96 program (Bio-Rad) in the exponential phase of amplification. A synthetic gene coding for the nucleotides 954 to 1306 of the MR766 strain (GenBank: LC002520) cloned in the pUC57 plasmid was used as template to generate a standard curve, which then served to make an absolute quantitation of the bound viruses.

### 4.9. Plaque Forming Assays

Plaque forming unit assays were used to quantify the release of infectious viral particles [23,28]. Vero cells were seeded the previous day in 48-well culture plates at a density of 3×10^4^ cells per well. Cells were infected by 0.1 mL of ten-fold dilutions of supernatant. Following an incubation of 2 h at 37 °C, 0.2 mL of the culture medium supplemented with 5% fetal bovine serum (FBS) and 0.8% carboxymethylcellulose sodium salt (Sigma-Aldrich, Saint-Quentin-Fallavier, France)) were added, and the incubation was extended for 4 days at 37 °C. Cells were fixed (PFA, 3.7%) and stained with 0.5% crystal violet (Sigma-Aldrich) diluted in 20% ethanol, after the media had been removed. Plaques were counted and expressed as plaque-forming unit per mL (PFU mL^−1^).

### 4.10. Foci-Forming Immunodetection Assay

C6/36 cells were seeded at a density of 1×10^5^ in 24 well plates and incubated overnight at 37 °C to perform a foci-forming immunodetection assay. Ten-fold serial dilutions of the supernatant were prepared in duplicate in the culture medium and 0.4 mL of each dilution was added to the cells for 1 h 30 min. Then, the inoculum was removed and a CMC media (L-15 supplemented with 10% FBS, 0.52% tryptose, 50 mg mL^−1^ gentamicin, 1.6% carboxymethylcellulose) was added. After seven days of being incubated, immunostaining was performed using the mouse monoclonal antibody 4G2 followed by goat-antimouse immunoglobulin conjugated to alkaline phosphatase (Promega, Madison, WI, USA). A solution of NBT (nitroblue tetrazolium chloride) and BCIP (5-bromo-4-chloro-39-indolyphosphate p-toluidine salt) (Promega, Madison, WI, USA) was used as a substrate to detect antibodies. The foci were counted and expressed as FFU mL^−1^.

### 4.11. Quantification of the Number of ZIKV-ssRNA Molecules by Fluorescence In Situ Hybridization (FISH) and Confocal Analysis

The ZIKV strains were pre-incubated for 1 h at 37 °C with plant extracts in DMEM before infection. *P. mauritianum* extract was used at a final concentration of 100 μg mL^−1^ and EGCG at a final concentration of 100 µM. Vero cells were then infected at MOI of 1 for 24 h with viruses treated or not with the drugs. After fixation with 4% PFA for 30 min at RT, the cells were stained with wheat germ agglutinin (WGA) AF-488 conjugate (Thermo Fisher Scientific, Waltham, MA, USA) in PBS-BSA 0.5% overnight at 4 °C. After immunostaining, ZIKV plus strand RNA was detected using Alexa-Fluor 546-conjugated probe sets recognizing the regions between nt 2 and 1144 of the ZIKV genome (Thermo Fisher Scientific). The samples were processed following the manufacturer ‘ViewRNA ISH Cells Assays’ protocol. The nuclei were stained with NucBlue (Thermo Fisher Scientific) for 15 min at RT. The images were acquired with a Zeiss LSM 700 laser scanning confocal microscope equipped with an X63 objective (Zeiss, Oberkochen, Germany). The images were processed with Zen2 (blue edition) software and analyzed with the ICY software (icy.bioimageanalysis.org) using the Spot Detector plugin. The number of individual RNAs per field was analyzed by assembling 3 × 3 images. A detection filter based on a size of 3 pixels in diameter (with a minimum of 2 pixels and a maximum of 5) was set up. Based on this size parameter, all the fluorescent signals, including ‘aggregated’ RNAs, were analyzed by the software and decomposed into individual RNA.

### 4.12. Data Analysis

Comparison between different concentrations was done by a one-way ANOVA test. All values were expressed as mean ± SD of at least three independent experiments. All statistical tests were done using the software Graph-Pad Prism (version 7.0; La Jola, CA, USA). Values of *p* < 0.05 were considered statistically significant for a Dunnett’s multiple comparisons test. Degrees of significance are indicated on the figure as follow: * *p* < 0.05; ** *p* < 0.01; *** *p* < 0.001, **** *p* < 0.0001, n.s = not significant.

## Supplementary Materials

Supplementary materials can be found at https://www.mdpi.com/1422-0067/20/8/1860/s1.

## Abbreviations

A549	Human lung epithelial cell line
CMC	Carboxymethyl cellulose
COMET	Single Cell gel electrophoresis
DENV	Dengue virus
DMSO	Dimethyl sulfoxide
DNA	Desoxyribonucleic Acid
EDTA	Ethylenediaminetetraacetic acid
EGCG	Epigallocatechin gallate
FACS	Fluorescence-activated cell sorting
GFP	Green fluorescent protein
HKPM	Primary human keratinocytes plasty mammary
hp.i	Hours post infection
Huh-7.5	Human hepatoma cell line
MOI	Multiplicity of infection
MTT	3-[4–dimethylthiazol-2-yl]-2,5-diphenyltetrazolium bromide
PBS	Phosphate-buffered saline
PFA	Paraformaldehyde
PFU	Plaque forming unit
RNA	Ribonucleic Acid
TRIS	Tris(hydroxymethyl)aminomethane
ZIKV	Zika virus
