# Supplementary material for: The Polyphenol-Rich Extract from Psiloxylon mauritianum, an Endemic Medicinal Plant from Reunion Island, Inhibits the Early Stages of Dengue and Zika Virus Infection
Source: Int J Mol Sci. 2019 Apr 15;20(8):1860. doi: 10.3390/ijms20081860 (PMC6515236; doi:10.3390/ijms20081860)
Supplement: Supplementary file 1 [file ijms-20-01860-s001.pdf]

Supplementary data

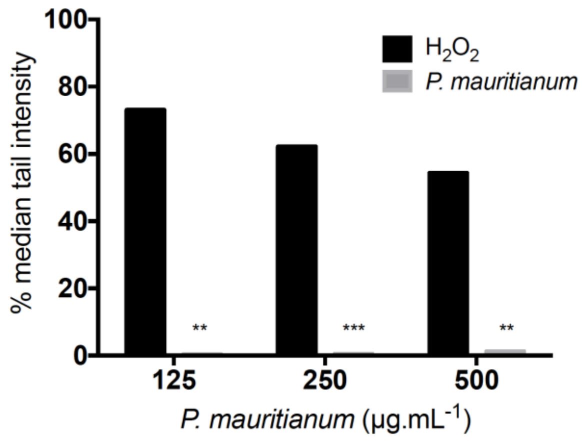

Figure S1: *P. mauritianum* extract do not exert genotoxic effect on human cells. A549 cells were treated 24h with different concentration of *P. mauritianum* extract. The genotoxicity was determined by observation of DNA degradation using COMET assay. Comet were visualized by Gel red and analyzed by COMET assay IV software. Results are means ± SD performed in triplicate and expressed as percentage of tail intensity. Statistical analyses were performed using two-way ANOVA and Sidak's multiple comparisons test (* $p<0.05$; ** $p<0.01$; *** $p<0.001$).

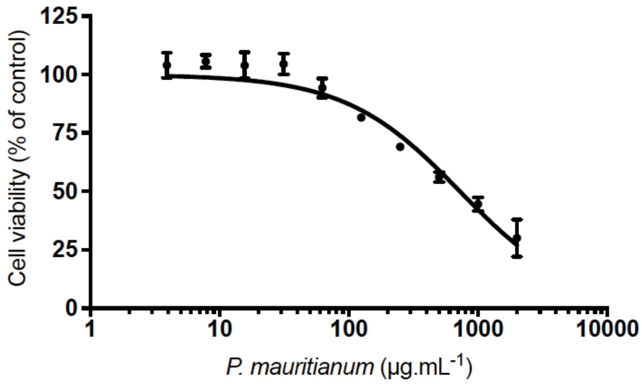

Figure S2: Cytotoxicity effect of *P. mauritianum* extract on Huh7.5 cells. Huh7.5 cells were treated 48h with different concentration of *P. mauritianum* extract. Cell viability was evaluated using MTT assay. Results are means ± SD of three independent experiments and are expressed as relative value compared to untreated cells.

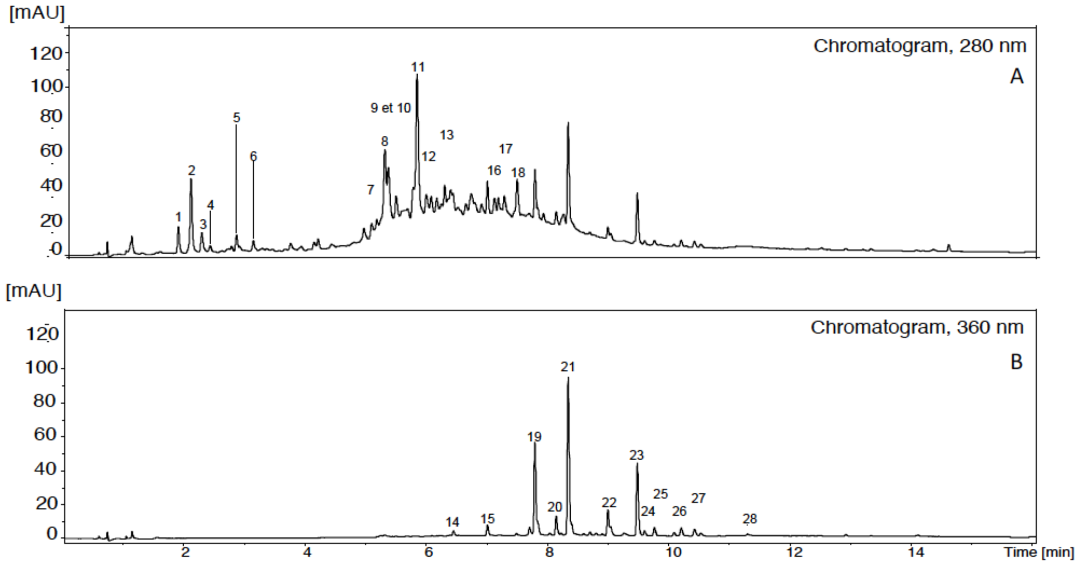

Figure S3: UHPLC-DAD chromatogram of *Psiloxylon mauritianum* extract at 280 nm (A) and 360 nm (B). *P. mauritianum* extract was characterized by UHPLC apparatus (Agilent 1290, Santa Clara, CA) equipped with an Agilent Zorbax SB-C18 (100 nm x 2.1 nm x 1.8 µm) column and an UV-VIS-DAD. The UHPLC apparatus was coupled with an Esquire 6000 ion trap mass spectrometer using an ESI source (Bruker Daltonics, Billerica, MA). Alternating negative and positive mode was performed for analysis. *P. mauritianum* extract was dissolved in MeOH-H_2O mixture (1/1, v/v) at 5 $mg.mL^{-1}$. Analyses were conducted in triplicate.

Table S1: Identification of polyphenols from *P. mauritianum* extract

Peaks	Compounds	t_R (min)	$[M-H]^-$ (m/z)	MS/MS fragments	Concentration w/w (in %)
1	Monogalloylhexoside(a)	1.9	331	169	1,011
2	Gallic acid*(a)	2.1	169	125	3,380
3	HHDP-galloylglucose(a)	2.3	633	482, 301	0,944
4	Digalloylhexoside(a)	2.4	483	331, 169	0.260
5	HHDP-galloylglucose(a)	2.9	633	481, 301	0.696
6	Digalloylhexoside(a)	3.1	483	331, 169	0.455
7	HHDP-digalloyl-glucose(a)	5.1	785	633, 483, 301	0.439
8	ni	5.3	843		nq
9	ni	5.4	859		nq
10	Trigalloylhexoside(a)	5.4	685	483, 465	nq
11	ni	5.8	859		nq
12	HHDP-digalloyl-glucose(a)	6.0	785	633, 483, 301	0.990
13	Trigalloylhexoside(a)	6.3	635	483,465	0.653
14	Myricetin-O-hexoside(b)	6.4	479	317	0.077
15	Quercetin-O-hexoside(b)	7.0	463	301	0.243
16	HHDP-trigalloyl-glucose(a)	7.1	937/469	785, 767, 465	0.608
17	HHDP-trigalloyl-glucose(a)	7.3	937/469	785, 767, 465, 447	0.476
18	Tétragalloylhexoside(a)	7.5	787	635, 617, 465	1.668

19	Quercetin-O-hexoside[(b)]	7.8	463	301, 179, 151	2.576
20	Kaempferol-O-hexoside[(c)]	8.1	447	285	0.427
21	Kaempferol-O-hexoside[(c)]	8.3	447	285	4.087
22	Quercetin-O-acetylhexoside[(b)]	9.0	505	463, 445, 301, 179, 151	0.704
23	Kaempferol-O-acetylhexoside[(c)]	9.5	489	447, 429, 285	1.946
24	Kaempferol-O-acetylhexoside[(c)]	9.6	489	429, 285	0.049
25	Quercetin-O-acetylrhamnoside[(b)]	9.8	489	447, 429, 301, 179, 151	0.201
26	Kaempferol-O-acetylrhamnoside[(c)]	10.2	473	413, 285	0.148
27	Kaempferol-O-acetylrhamnoside [(c)]	10.4	473	413, 285	0.156
28	Kaempferol*[(c)]	11.3	285		nq

ni: not identified; nq: not quantified.

* Retention time verified with commercial standard

(a) quantified as gallic acid equivalent at 280 nm

(b) quantified as quercetin equivalent at 360 nm

(c) quantified as kaempferol equivalent at 360 nm
